# One Way to Design a Valence-Skip Compound

**DOI:** 10.1186/s11671-017-1897-z

**Published:** 2017-02-17

**Authors:** I. Hase, T. Yanagisawa, K. Kawashima

**Affiliations:** 1 National Institute of Advanced Industrial Science and Technology (AIST), AIST Central 2, Umezono 1-1-4, Tsukuba, Ibaraki 305-8568 Japan; 2 IMRA Material R&D Co., Ltd., Kariya, Aichi 448-0032 Japan

**Keywords:** Valence skip, CDW, Superconductivity, Electronic structure, RbTlCl_3_, BaBiO_3_

## Abstract

Valence-skip compound is a good candidate with high *T*_c_ and low anisotropy because it has a large attractive interaction at the site of valence-skip atom. However, it is not easy to synthesize such compound because of (i) the instability of the skipping valence state, (ii) the competing charge order, and (iii) that formal valence may not be true in some compounds. In the present study, we show several examples of the valence-skip compounds and discuss how we can design them by first principles calculations. Furthermore, we calculated the electronic structure of a promising candidate of valence skipping compound RbTlCl_3_ from first principles. We confirmed that the charge-density wave (CDW) is formed in this compound, and the Tl atoms in two crystallographic different sites take the valence Tl^1+^ and Tl^3+^. Structure optimization study reveals that this CDW is stable at the ambient pressure, while this CDW gap can be collapsed when we apply pressure with several gigapascals. In this metallic phase, we can expect a large charge fluctuation and a large electron–phonon interaction.

## Background

In order to utilize superconducting materials, essentially three properties are highly desirable: high critical temperature (*T*_c_), low anisotropy, and good workability. However, the materials satisfying these three conditions are not found at present. For example, cuprates have high *T*_c_, but their anisotropy is extremely high, because of their two-dimensional nature and the origin of superconductivity, i.e., strong repulsive Coulomb’s interaction of the conduction electrons. Superconducting gap equation requires that if the sign of the interaction is positive; then, the superconducting gap must have a node, eventually this gap must be anisotropic.

If we can use an attractive interaction as a glue of superconductivity (SC), the appeared SC might be isotropic. Phonon-mediated SC is one of them, while its *T*_c_ is limited by the Debye temperature. In 1988, Varma proposed a new mechanism of SC, which uses the valence-skip element [1]. For example, Bi takes 3+ or 5+ valence state in a compound and usually does not take 4+ valence state. We call this valence skip because it skips the 4+ valence state. In other words, the electron configuration of the outermost *s* orbitals forms a closed shell in most compounds, for example, in the case of Bi, s2 (completely filled) for Bi^3+^ and s0 (empty) for Bi^5+^. If we *force* the valence state of Bi as 4+ in a compound or the occupation of the outermost s-electrons as half-filled (s1), it has a large charge fluctuation. This charge fluctuation is attractive one because the effective Coulomb interaction *U*_eff_ = *E*(Bi^3+^) + *E*(Bi^5+^)−2*E*(Bi^4+^) becomes negative, where *E*(Bi^*n*+^) denotes the energy of Bi n+ state. If this type of compound becomes superconducting (in fact, there are a few examples), it might become a good material for applications.

However, in most cases, this fluctuation is frozen and forms a charge order or so-called charge-density wave (CDW). For example, BaBiO_3_ shows CDW in the low temperature phase [2]. In this phase, the crystal symmetry lowers due to the displacement of the oxygen atoms. Bi occupies two crystallographic sites, one is Bi^3+^ and the other is Bi^5+^. This frozen order can be melt by two methods: First, by raising temperature, since high temperature disfavors the ordered state. This is the thermal effect and rather trivial. Second, by carrier doping in the potassium-doped (Ba,K)BiO_3_, the charge order is melt and the high-symmetry of the crystal structure is restored. Quite interestingly, (Ba,K)BiO_3_ shows SC below *T*_c_ = 39 K [3].

Our goal is to synthesize new superconductors, those are caused by the valence-skip mechanism. The first step to this goal is very simple, just “search s1 compound.” The importance of s1 configuration of the cation is also emphasized by the discoverer of the abovementioned BaBiO_3_, A. W. Sleight [4]. In fact, we can survey a database such as Pearson’s Crystal Data [5] and find some s1 compounds. However, this is not enough by the following reasons: Firstly, a compound which formally has s1 configuration does not necessary contain one electron in the outermost *s* orbital of the cation. Secondly, many s1 compounds show CDW, and this CDW is too hard to be melt by doping. First principles calculation can shed light to these problems. Recently, along this strategy, the electronic structure of ATlX_3_ (*A* = Rb, Cs; *X* = F, Cl, Br) was investigated [6]. As expected, the electronic structure of ATlX_3_ and BaBiO_3_ quite resemble each other. They both have a charge order of (Tl^1+^, Tl^3+^) or (Bi^3+^, Bi^5+^), accompanied with the breathing mode of anions. In general, insulators can be metallic by two different ways: One way is the carrier doping, mostly done by substituting a different valence ion. The other way is applying pressure. The latter method has a merit that it does not create randomness, which may degrade or deteriorate the superconductivity. Unfortunately, probably since BaBiO_3_ is too hard to compress, the pressure-induced superconductivity has not been realized yet. In this paper, we show that RbTlCl_3_ can be metallic by applying a tractable pressure. Considering that the electronic structure of RbTlCl_3_ is similar to that of BaBiO_3_, we can also expect superconductivity in RbTlCl_3_ under pressure.

This paper is organized as follows: In the “Methods” section, the crystal structures of the compound that we calculated and the method of calculation are described. In the “Results and Discussion” section, we introduce our surveying work for searching for the valence-skip compound. Firstly, we discuss some relationships between the valence, s-p energy difference, and charge fluctuation for some compounds which formally have s1 configuration. Secondly, we report the possibility of pressure-induced valence-skip superconductivity in RbTlCl_3_. Summary is described in the “Conclusions” section.

## Methods

### Crystal Structure

It is known that CsTlCl_3_ has the ordered perovskite structure in the high temperature phase (it is written as elpasolite in the literature) [7]. Although RbTlCl_3_ is not synthesized yet, it is highly plausible that these compounds have the same structure because they are isovalent and the tolerance factor is around ~1 [6]. In the present work, we ignore the possible small tilting distortion of anions and concentrate on the ordered perovskite phase for simplicity. In this structure, Tl^1+^ and Tl^3+^ ions are alternatively order like NaCl and the X atoms move toward Tl^3+^ because of the larger positive charge and/or smaller ionic radius of Tl^3+^. This displacement of *X* atom is the same with the breathing mode of BaBiO_3_. The crystal structure of BaBiO_3_ and RbTlCl_3_ is as follows: Space group Fm-3m (#225), Ba/Rb (1/4,1/4,1/4), Bi1/Tl1 (0,0,0), Bi2/Tl2 (1/2,1/2,1/2), and O/Cl (0,0,*z*). When *z* = 0.25, the anion is located at the midpoint of two Bi/Tls and the crystal structure becomes the simple perovskite one. For BaBiO_3_, we used *z* = 0.25 since we are interested in the metallic state and for RbTlCl_3_, we fully relaxed the parameter *z* by total energy minimization. For both compounds, we ignored the small monoclinic distortion for simplicity.

### Method of Calculations

We have calculated the electronic structure of RbTlCl_3_ from first principles. We have used a full potential augmented plane wave (FLAPW) scheme, and the exchange-correlation potential was constructed within the general gradient approximation [8]. Hereafter, we call this potential as PBE. We used the computer program WIEN2k package [9]. The parameter *RK*_max_ is chosen as 7.0. The k-point mesh is taken so that the total number of mesh in the first Brillouin zone is ~1000. We have also optimized the crystal structure, with fixing the space group and the lattice parameter. In this structure, the only one free parameter is the position of Cl(=*z*). The convergence of atomic position is judged by the force working on each atom is less than 1.0 mRy/a.u. As for BaBiO_3_ and RbTlCl_3_, we also used KANSAI94 program set in order to compare with the previous results of InTe, SnAs, and PbSb [10]. Here, we used the local density approximation for the exchange-correlation potential [11]. We set the muffin-tin radii of BaBiO_3_ as *r*(Ba) = 2.6 a.u., *r*(Bi) = 2.5 a.u., and *r*(O) = 1.6 a.u. As for RbTlCl_3_, we set the muffin-tin radii as *r*(Rb) = 2.6 a.u., *r*(Tl) = 2.5 a.u., and *r*(Cl) = 2.2 a.u. The calculated band structures using WIEN2k and KANSAI94 are almost the same as is expected.

### Tight-Binding Analysis and Estimation of Valence State

In order to obtain more insight of the electronic structure of these compounds, we fit the *E*(*k*) curve by the following tight-binding (TB) model Hamiltonian:

1 $$ {\widehat{H}}_0={\displaystyle \sum_i{\varepsilon}_i^{\mu}}{c}_{i\mu}^{\dagger }{c}_{i\mu}+{\displaystyle \sum_{i j}{t}_{i j}^{\mu \nu}}{c}_{i\mu}^{\dagger }{c}_{j\nu} $$

Here, ${\varepsilon}_i^{\mu}$ denotes the on-site energy of *i*-site with *μ* orbital (*μ* = *s*, *p*) and ${t}_{ji}^{\mu \nu}$ denotes the transfer matrix element between the *μ* orbital in *i*-site and the *ν* orbital in *j*-site. We omit the spin indices for simplicity. We only consider the *s* and *p* orbitals both for cation and anion and only consider the nearest neighbor hopping. As for the NaCl-type compounds (InTe, SnAs, and PbSb), we already discussed precisely in the previous paper [10]. As for the perovskite compounds (BaBiO_3_ and RbTlCl_3_), we assumed *z* = 0.25 (i.e., no distortion) and adopted the five-parameter model which is used in Ref. [12].

In order to estimate the valence state, we calculate two quantities: One is the number of occupied s-electron in the cation muffin-tin sphere (=*N*_s_mt_), and the other is the number of occupied s-electron within a *s* and *p* orbitals tight-binding model used in Ref. [10] (=*N*_s_tb_). Both quantities have advantages and disadvantages. *N*_s_mt_ can be directly calculated by this first principles calculation, but it depends on the muffin-tin radius. And since these compounds do not include *d* or *f* orbitals in the valence bands, the wave function spreads out to the interstitial region. Thus, the estimation of the valence state of cation is not straightforward. On the other hand, since *N*_s_tb_ is based on the tight-binding model, it does not directly depend on spread of the wave function. And when we include many-body effect (e.g., negative-*U* Hubbard model), tight-binding basis is necessary. However, tight-binding fitting to the first principles results causes ambiguity. Therefore, *N*_s_mt_ and *N*_s_tb_ are complimentary to each other.

## Results and Discussion

### s1 Compound or not?

**Fig. 1:**
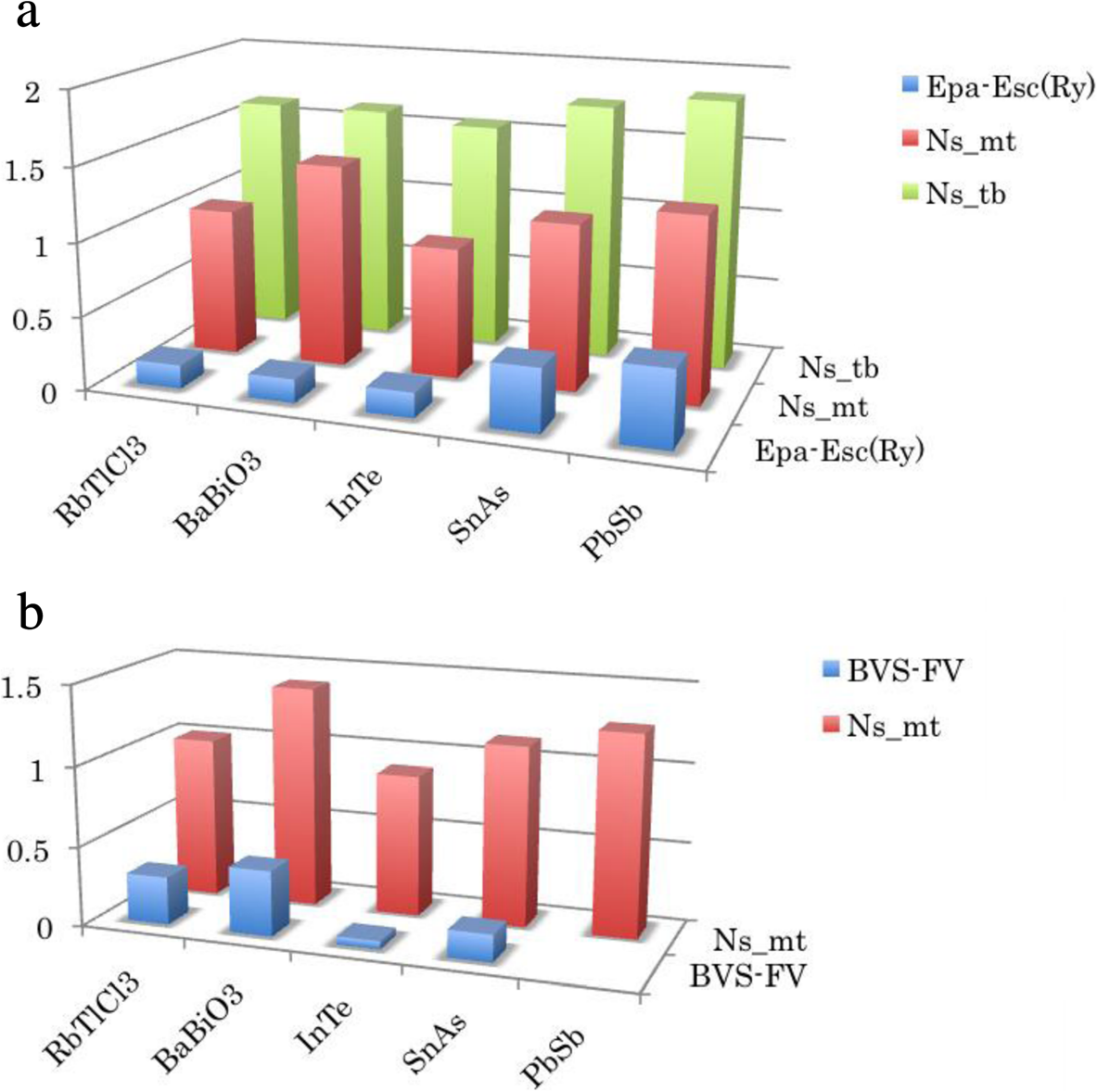
**a** Characteristic parameters for BaBiO_3_ and three binary formally s1 compounds. **b** The difference between the bond valence sum and the formal valence (BVS-FV) compared with *N*_s_mt_. Note that we do not find the bond valence sum of PbSb because there is no data of Pb-Sb bond parameter in the literature [16]

In the preceding paper, we have calculated the band structures of three binary compounds InTe, SnAs, and PbSb with rocksalt structure [10]. In order to obtain the information of the valence of these compounds, we have performed a systematic tight-binding analysis based on ab-initio calculation. Figure 1a shows the *N*_s_mt_, *N*_s_tb_, and *E*_p_^a^−*E*_s_^c^ (energy difference between anion *p* orbital and cation *s* orbital in the tight-binding model) for five valence-skip candidate compounds. We can see a clear tendency that *N*_s_tb_ increases when *E*_p_^a^−E_s_^c^ increases. Moreover, InTe has almost the same value of *N*_s_tb_ and *E*_p_^a^−*E*_s_^c^ with BaBiO_3_, indicating that in InTe the In atom behaves as the Bi atom in BaBiO_3_. In fact, in the ambient pressure phase, InTe shows CDW, similar to BaBiO_3_. Therefore, we can say that InTe has s1 configuration, in the sense that BaBiO_3_ apparently has s1 configuration. RbTlCl_3_ has almost the same values of *N*_s_tb_ and *E*_p_^a^−*E*_s_^c^, so we conclude that RbTlCl_3_ also has s1 configuration. We can also claim that PbSb is *not* a valence skipper because *N*_s_tb_ is near two. The strength of the valence-skip fluctuation is strong in InTe and BaBiO_3_ and is weak in PbSb. From these results, we can explain why PbSb does show neither CDW nor SC, and why InTe and BaBiO_3_ show CDW at ambient pressure and show SC by some perturbation (pressure in InTe and doping in BaBiO_3_).

We found that *N*_s_mt_ shows an abnormal behavior in BaBiO_3_ and RbTlCl_3_, while in other three binary compounds, *N*_s_mt_ has the same tendency as *N*_s_tb_. The muffin-tin radii of cations are also set as 2.5 a.u. for all these compounds, and we also have checked that the decrease of the cation muffin-tin sphere gives almost the same ratio of *N*_s_mt_ decrease, i.e., *d*(*N*_s_mt_)/*d*(rmt) = 0.7~0.9/a.u. At present time, we do not find the clear reason why only BaBiO_3_ and RbTlCl_3_ have different tendency, i.e., too much *N*_s_mt_ compared to *N*_s_tb_. We consider that the difference of the crystal structure (perovskite vs. rocksalt) causes this different tendency of *N*_s_mt_. In order to see this, we have calculated the bond valence sum and compared with *N*_s_mt_ in Fig. 1b. Interestingly, the difference between the bond valence sum and the formal valence (=BVS-FV) is well correlated with *N*_s_mt_ for all these compounds. And we can see that InTe has almost In^2+^ valence state, and SnAs has a small discrepancy from Sn^3+^ valence state as we expected. On the contrary, BaBiO_3_ (RbTlCl_3_) shows a large discrepancy from Bi^4+^ (Tl^2+^) valence state and this behavior looks like that of *N*_s_mt_. Considering that the bond valence sum is sensitive to crystal structure, the anomalous behavior of *N*_s_mt_ in BaBiO_3_ and RbTlCl_3_ may come from the difference of the crystal structure.

### Pressure-Induced Metallic State in RbTlCl_3_

**Fig. 2:**
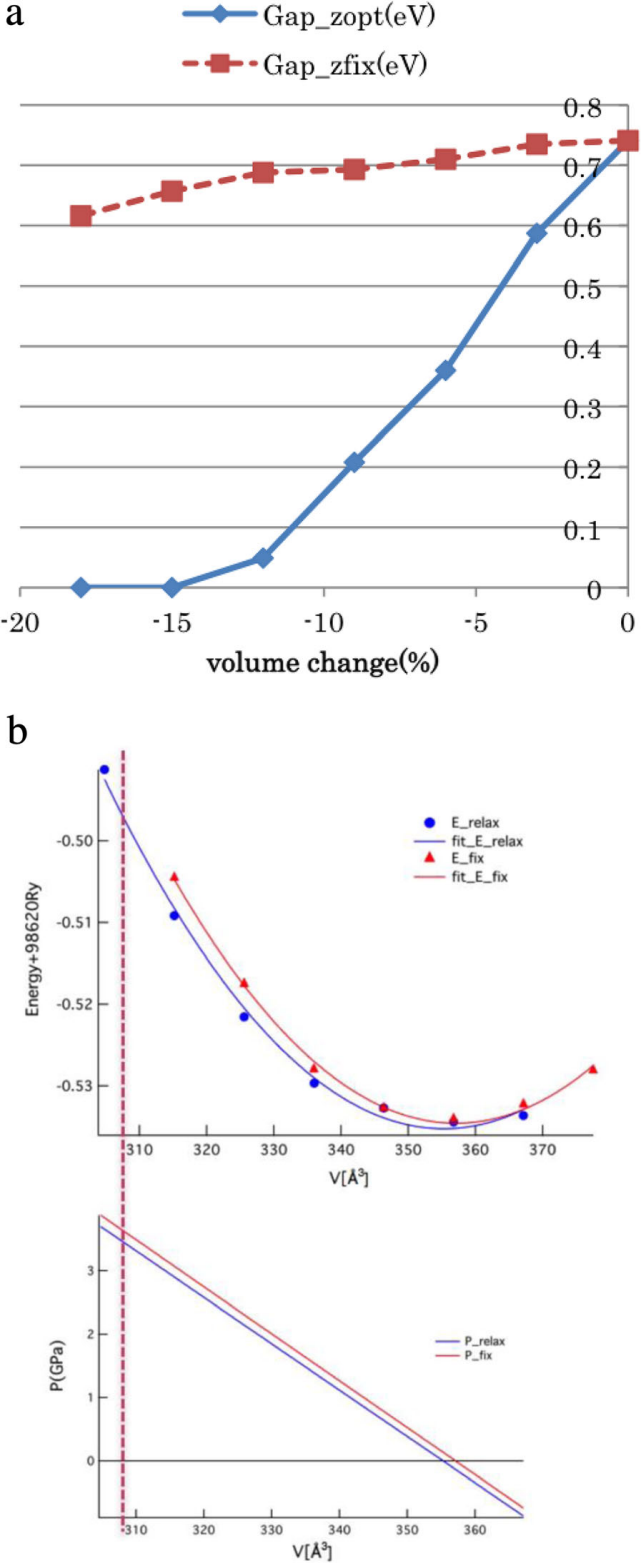
**a** The band gap of RbTlCl_3_ as a function of volume reduction. Δ*V* = 0 denotes the volume at ambient pressure. **b** The total energy and pressure of RbTlCl_3_ for various unit cell volume. Note that we set the formula unit as Rb_2_Tl^1+^Tl^3+^Cl_6_, which is twice of that in Ref. [6]. For both panels, the *dotted line* is for *z* = *z*_0_ (fixed) and the *solid line* is for optimized *z* for each volume

In order to determine whether RbTlCl_3_ shows CDW or not, we performed a structure optimization study by minimizing the total energy. In the ordered perovskite structure, the only two tunable structure parameters are the lattice parameter *a* (or the volume of the unit cell *V*) and the parameter *z* which denotes the position of Cl(0,0,*z*). At ambient pressure (determined by the energy minimization), *a*_0_ = 11.254 Å and *z*_0_ = 0.2362 are obtained. The obtained lattice parameter *a*_0_ is close to the value of the previous work [6]. If *z* becomes 0.25, then Cl atom locates at the midpoint of Tl1 and Tl2 atoms, crystal symmetry becomes higher and CDW disappears. Our result *z*_0_ = 0.2362 shows that CDW occurs in RbTlCl_3_ at ambient pressure. The band gap is opened between the Tl1_s band and the Tl2_s band, similar to the case of SnF_3_ and BaBiO_3_ [13]. Therefore, RbTlCl_3_ is a typical valence skipper. When we choose some *a* and *z*, the band gap Δ is determined. In other words, Δ is a function of *a* and *z*. Figure 2 shows this Δ as a function of *V* = (*a* ^3^/4). The dotted line shows Δ when *z* is fixed to *z*_0_ = 0.2362 (*z* value at ambient pressure). We can see that Δ is rather insensitive to *V* and one might think it is impossible to collapse the gap by pressure. However, the optimized *z* is different for each *V* and we show the *V*-dependence of Δ with optimizing *z* in the solid line of Fig. 2. Applying pressure not only decreases *V* but also change *z* along *V*-dependence of *z*. Therefore, we can control *z* via changing *V* and eventually collapse the gap. We can also describe the *V* dependence of the total energy in the same way, shown in Fig. 2b. These data can be fit by the Murnaghan equation of state curve. Again, the dotted line denotes *E*(*V*) when *z* is fixed to *z*_0_ and the solid line denotes *E*(*V*) with optimizing *z* for each *V*. The vertical line corresponds to the critical volume *V*_c_, defined as Δ(*V*_c_) = 0. Since the obtained bulk modulus *B* is very small (=26 GPa), the corresponding pressure is only ~3 GPa. Considering that density functional calculation usually underestimates the value of the band gap, applying pressure with 4~5 GPa may be enough to collapse the band gap. In order to check this point, we performed a test calculation using so-called mBJ potential functional [14], which can improve the value of the band gap [15]. As for *V* = *V*_0_ (ambient pressure), the magnitude of the band gap is 1.27 eV using mBJ and 0.74 eV using PBE. On the other hand, at *V* = 0.85 *V*_0_, the band gap diminishes both for mBJ and PBE. Therefore, we conclude that the metal-insulator transition occurs even we use mBJ potential functional. Since we do not optimize *z* for mBJ calculation, the critical volume *V*_c_ and critical pressure may change. The resulting metallic phase should have a large charge fluctuation and a large electron–phonon interaction, which is quite advantageous for isotropic superconductivity.

## Conclusions

In order to search the valence-skip superconductor, we investigated the electronic structure of several compounds formally having s1 configuration by first principles study. Nevertheless, combined with a tight-binding analysis, we found that some compounds have almost s2 configuration. We also found that RbTlCl_3_ is a good candidate of the pressure-induced superconductor, with having a large charge fluctuation and electron–phonon interaction.

## Abbreviations

CDW: Charge-density wave
FLAPW: Full-potential augmented plane wave
mBJ: Modified Becke-Johnson
PBE: Perdew-Burke-Ernzerhof
SC: Superconductivity
*T*_c_: Critical temperature

## References

[1] Varma CM (1988). Phys Rev Lett.

[2] Sleight AW, Gilson JL, Bierstedt PE (1975). Solid State Commun.

[3] Mattheiss LF, Gyorgy EM, Johnson DW (1988). Phys Rev.

[4] Sleight AW (2015). Physica.

[5] Pearson’s Handbook Desk Edition, ASM International (1997).

[6] Schoop LM, Müchler L, Felser C, Cava RJ (2013). Inorg Chem.

[7] Ackermann R, (2001) Thesis, Universitätsbibliothek Freiburg, Breisgau

[8] Perdew JP, Burke K, Ernzerhof M (1996). Phys Rev Lett.

[9] Blaha P, Schwarz K, Madsen GKH, Kvasnicka D, Luitz J (2001). WIEN2k, an augmented plane wave + local orbitals program for calculating crystal properties.

[10] Hase I, Yasutomi K, Odagiri K, Yanagisawa T, Nishio T (2016). Physica.

[11] Gunnarson O, Lundqvist BI (1976). Phys Rev.

[12] Mattheiss LF, Hamann DR (1983). Phys Rev.

[13] Hase I, Yanagisawa T, Kawashima K (2016). Phys C Phys.

[14] Tran F, Blaha P (2009). Phys Rev Lett.

[15] Camargo-Martinez J. A., Baquero R. (2012) Phys. Rev. B86 195106 ; Rev. Mex. Fis. 59 (2013) 453.

[16] Brown ID, Altermatt D (1985). Acta Cryst.

